# Temperature affected guided wave propagation in a composite plate complementing the *Open Guided Waves* Platform

**DOI:** 10.1038/s41597-019-0208-1

**Published:** 2019-10-04

**Authors:** Jochen Moll, Christian Kexel, Serena Pötzsch, Marcel Rennoch, Axel S. Herrmann

**Affiliations:** 1 Goethe University of Frankfurt, Department of Physics, Max-von-Laue-Straße 1, 60438 Frankfurt am Main, Germany; 2 Faserinstitut Bremen e. V. (FIBRE), Am Biologischen Garten 2, 28359 Bremen, Germany

**Keywords:** Mechanical engineering, Design, synthesis and processing

## Abstract

The influence of temperature is regarded as particularly important for a structural health monitoring system based on ultrasonic guided waves. Since the temperature effect causes stronger signal changes than a typical defect, the former must be addressed and compensated for reliable damage assessment. Development of new temperature compensation techniques as well as the comparison of existing algorithms require high-quality benchmark measurements. This paper investigates a carbon fiber reinforced plastic (CFRP) plate that was fully characterized in previous research in terms of stiffness tensor and guided wave propagation. The same CFRP plate is used here for the analysis of the temperature effect for a wide range of ultrasound frequencies and temperatures. The measurement data are a contribution to the *Open Guided Waves* (OGW) platform: http://www.open-guided-waves.de. The technical validation includes initial results on the analysis of phase velocity variations with temperature and exemplary damage detection results using state-of-the-art signal processing methods that aim to suppress the temperature effect.

## Abstract (summary)

**Table:** 

Measurement(s)	Guided ultrasound waves in a composite plate under temperature variations • temperature
Technology Type(s)	Piezoelectric transducers • Temperature Sensor Device
Factor Type(s)	excitation frequency • temperature

Machine-accessible metadata file describing the reported data: 10.6084/m9.figshare.9863465

## Background & Summary

The application of guided ultrasound waves for non-destructive testing (NDT) and structural health monitoring (SHM) applications is complex due to their multimode and frequency-dependent behaviour^1,2^. Changes in the structure due to structural damage such as a crack, delamination or corrosion damage should be detected while ambient influences such as temperature variations should be compensated.

The temperature effect has a significant influence on the ultrasound signals given by the thermal expansion of the structure as well as temperature-induced wave velocity changes^3^. A long-time stability analysis of electromagnetic acoustic transducers over a period of 14 months was performed in^4^. It was found that changes in temperature alter the waves amplitude and phase. To separate the effect of varying temperature on propagating guided waves from their scattering at defects, several temperature compensation methods have been proposed in the literature^5–12^. However, the geometry and material to which the methods have been applied differ strongly from each other. This makes an overall quality assessment of the algorithms performance challenging.

Based on this observation, the present paper aims to complement the Open Guided Waves repository using benchmark datasets obtained from a carbon fiber reinforced plastic (CFRP) plate under varying temperature conditions. The measurement data contain a large number of excitation frequencies for all actuator-sensor combinations. This enables a performance analysis using existing temperature correction algorithms and the development of novel temperature compensation methodologies. The unique property of the proposed CFRP plate is the detailed previous characterization in terms of stiffness tensor and guided wave propagation^13^. The available datasets at room temperature are now extended by additional controlled temperature experiments in a climate chamber ranging from 20 °C to 60 °C with a 50% RH (relative humidity).

The technical validation of the proposed datasets include a number of representative results using state-of-the-art signal processing techniques. The results demonstrate the plausibility of the measurements and give an impression on the possible re-use, addressing the importance and necessity of benchmark data.

## Methods

**Fig. 1:**
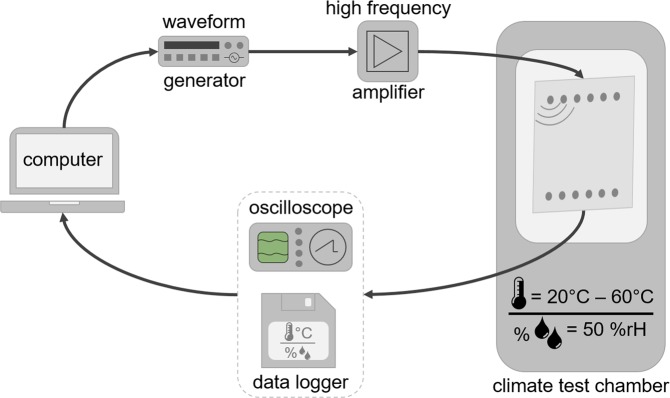
Scheme of the experimental setup. It illustrates the devices for measurement and detection and indicates the propagating guided ultrasonic waves in the specimen with indication of the 12 piezoelectric discs, placed in a climate test chamber.

Figure 1 illustrates a scheme of the experimental setup, indicating the measurement devices and the specimen placed in a climate test chamber. The device under test is a composite plate made from HexplyⓇ M21/34%/UD134/T700/300 carbon pre-impregnated fibers. The plate has a quasi-isotropic layup with a stacking sequence of [45/0/−45/90/−45/0/45/90]_*S*_ and its dimension is 500 mm × 500 mm with a thickness of 2 mm. The mechanical properties of the plate are documented in^13^. DuraAct piezoelectric transducers are co-bonded to the plate during the curing process in the autoclave. Each piezoelectric disc measures 0.2 mm in thickness and 10 mm in diameter. The arrangement and the coordinates of the piezoelectric transducers are listed in Table 1. A Handyscope HS5 (TiePie Engineering) is used to generate arbitrary waveforms and to record the signals by analog-to-digital conversion (14 bit resolution). Next, a broadband amplifier PD200 (PiezoDrive Ltd.) amplifies the excitation waveforms and forwards the signals to a custom multiplexer described in^14^. The latter device allows measuring all actuator-receiver combinations by time-division multiplexing. The hardware also supports measurements of high frequencies (up to 1 MHz) and high currents (up to 2 A). In addition, two temperature probes measure the surface temperature of the plate in the upper and lower left corners. In this work, we also use the same reversible damage model as in the previous study: the damage is represented by an aluminum disc mounted on the surface of the specimen by tacky tape^15^. Although this reference damage is simplified with respect to the geometry of an actual delamination, its interaction with guided elastic waves is similar in terms of important ultrasound features like changes in time of flight (TOF) or decrease in amplitude as quantified in^16^. The damage model can easily be removed and placed on other positions on the structure without permanently damaging it. Here, we have selected a subset of possible damage positions as listed in Tables 2, i.e. *D*_04_, *D*_12_, *D*_16_ and *D*_24_. Please note that the damage positions are on a straight line which potentially could be of interest for TOF analysis.

**Table 1:** Coordinates of piezoelectric transducers on the plate.

Transducer ID	x-coordinate (mm)	y-coordinate (mm)
1	450	470
2	370	470
3	290	470
4	210	470
5	130	470
6	50	470
7	450	30
8	370	30
9	290	30
10	210	30
11	130	30
12	50	30

**Table 2:** Coordinates of damage position (center position of the circular disc).

Damage ID	x-coordinate (mm)	y-coordinate (mm)
*D*_04_	65	400
*D*_12_	195	330
*D*_16_	335	260
*D*_24_	450	190

**Table 3:** Description of attributes in h5-files.

Attribute	Description
CTC/Humidity	CTC stands for climate temperature chamber. The CTC/humidity value is the relative humidity (RH) in percent measured by the CTC
CTC/Temperature	The CTC/Temperature value is the temperature in degree Celcius measured by the CTC
Temperature/values	An array with two elements, where the first entry describes the surface temperature at the bottom left of the CFRP plate, and the second entry the surface temperature on the top left
command/pitchcatch/channels	This attribute describes the index of the transducer pair starting from zero, i.e. T1 has the value of 0
command/pitchcatch/sampling_frequency	Sampling frequency of the analog-to-digital converter (ADC), i.e. 10 MSPS
command/pitchcatch/signal_cycles	Number of cycles in the actuation signal, i.e. 5
command/pitchcatch/signal_data	Excitation waveform
command/pitchcatch/signal_frequency	Carrier frequency of the excitation pulse
pitchcatch/catch	Array containing the catch signals (row-wise)
pitchcatch/pitch	Array containing the pitch signals (row-wise)
timestamp	timestamp of the measurement

**Fig. 2:**
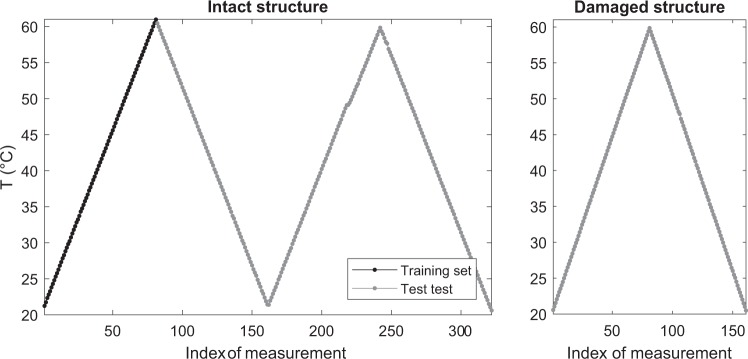
Temperature profile for the measurements. On the left two cycles for the intact structure and on the right one cycle for the damaged structure.

The excitation signal is a 5-cycle Hann-filtered sine wave amplified to ±100 V. All actuator-sensor pairs are measured in a round robin fashion for the frequency range from 40 kHz to 260 kHz in steps of 20 kHz. At the same time, the structure was exposed to temperature cycles ranging from 20 °C to 60 °C in steps of 0.5 °C as shown in Fig. 2. At each temperature level, all stated frequencies are recorded. Two temperature cycles were measured for the intact structure and one temperature cycle for each simulated damage position. Finally, all datasets were saved in a HDF5 container file format.

## Data Records

Recently, the Open Guided Waves platform was established where high quality benchmark measurements for guided wave-based NDT and SHM can be freely accessed online: www.open-guided-waves.de. The main goal of the OGW platform is to support research and developments in the field of guided wave technology. The aim of the present work is to support research in the area of temperature analysis and compensation techniques for improved diagnostic accuracy of guided wave-based systems.

The measured datasets^17^ are organized in five folders:

- OGW_CFRP_Temperature_udam: Baseline measurements of the intact plate
- OGW_CFRP_Temperature_dam_D24: Damage position is *D*_24_
- OGW_CFRP_Temperature_dam_D16: Damage position is *D*_16_
- OGW_CFRP_Temperature_dam_D12: Damage position is *D*_12_
- OGW_CFRP_Temperature_dam_D04: Damage position is *D*_04_.

Each folder consists of many subfolders, where the name of the subfolder indicates the time stamp in the format Year-Month-Day-Delimiter-Hours-Minutes-Seconds (e.g. 20181213T095826). Each subfolder contains a number of h5-files where the filename describes the current carrier frequency of the excitation toneburst (e.g. pc f100kHz.h5 where pc stands for pitch-catch mode. A description of the attributes in h5 files are listed in Table 3).

As a starting point, it is recommended to use a HDF5-viewer, such as HDFView https://support.hdfgroup.org/products/java/hdfview/, HDF Compass (https://github.com/HDFGroup/hdf-compass), or MATLAB command h5disp to open a h5-file and to see how the h5-files are organized. Table 2 provides detailed information about all HDF5 file attributes.

## Technical Validation

### Analysis of temperature-dependent phase velocity changes

Figure 2 depicts the temperature profile of the measurements for the intact and the damaged structure. It can be seen that the intact structure was exposed to two temperature cycles in the temperature range from 20 °C to 60 °C. On the other hand, a single temperature cycle was considered for each state of the damaged structure. The first 81 measurements of the intact structure served as a training set (for the Optimal Baseline Selection (OBS) procedure discussed below), while the remaining measurements were used as test set.

**Fig. 3:**
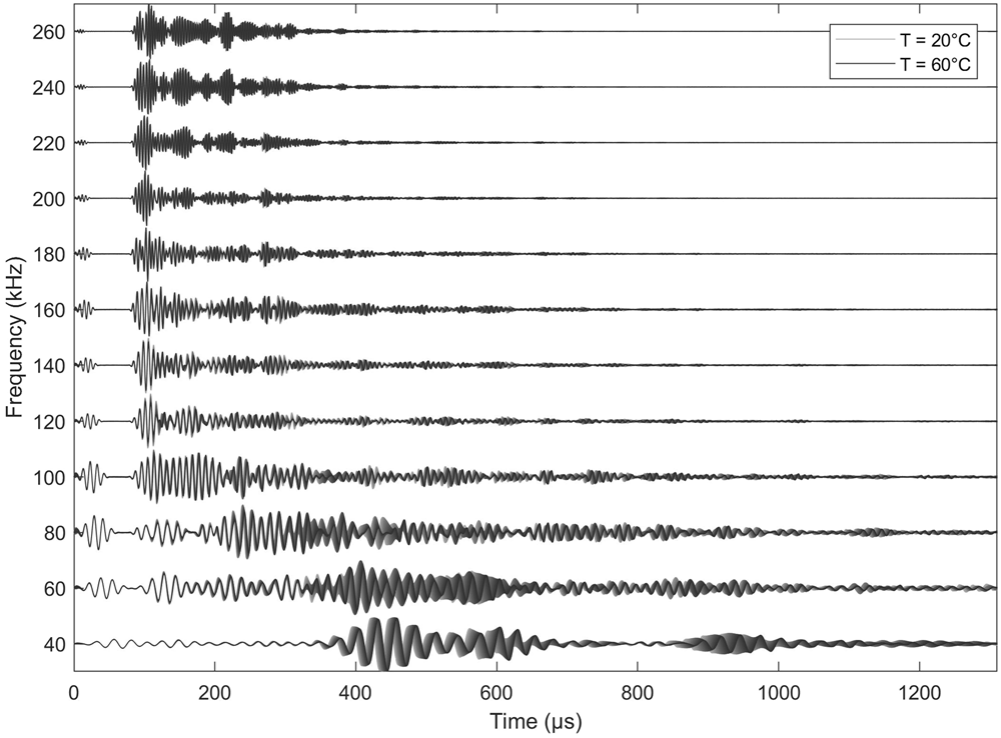
A-scan representation for the analysis of the temperature effect at different excitation frequencies and temperature levels (here: actuator *T*_4_ and receiver *T*_10_).

A large number of ultrasound signals for several excitation frequencies and temperature levels are shown in Fig. 3. The temperature information is color-coded from light gray representing a measurement at 20 °C to dark gray representing a measurement at 60 °C. This example is based on the actuator-sensor pair *T*_4_ − *T*_10_ and is representative for all other transducer combinations. While electromagnetic crosstalk can be observed at the beginning of each signal, the wave modes arrive at the sensor after a characteristic TOF. All signals have a high complexity due to the interference of multiple wave modes reflected from the edges of the structures. At lower frequencies the fundamental antisymmetric wave mode has a higher relative amplitude compared to the fundamental symmetric wave mode. This ratio reverses towards higher ultrasound frequencies due to the mode-tuning effect^18,19^.

Interesting aspects can be derived from this representation regarding the temperature dependency of guided wave modes. Both fundamental wave modes are affected by temperature causing phase and amplitude variations. Although the temperature effect is visually more pronounced for the antisymmetric wave mode, it can also be observed for the symmetric wave mode^3^. The physical interpretation is based on a temperature-dependent Young’s modulus which has been investigated in^20,21^. For an aluminum plate the Young’s modulus decreases with temperature in the order of −0.04 GPa/°C. Since the Young’s modulus is related to the wave velocity of the guided wave modes, a drop in velocity could be expected towards higher temperatures^22^.

**Fig. 4:**
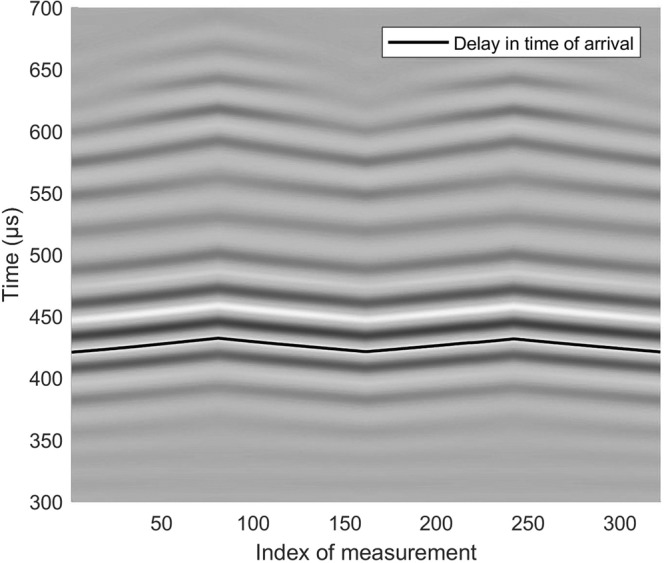
Waterfall diagram showing the temperature-induced variations in time of flight (TOF) for the actuator-receiver pair *T*_4_ − *T*_10_ at 40 kHz.

**Fig. 5:**
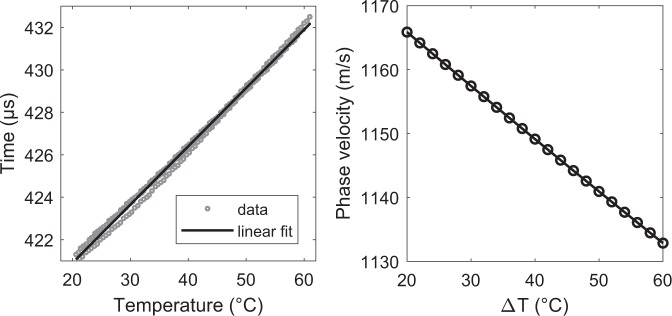
Changes in time of flight as a function of temperature (here: actuator *T*_4_ and receiver *T*_10_). The slope of the linear fit is 0.2749 *μs*/°C and the change rate of the phase velocity with temperature is −0.825 m/s/°C.

A quantitative analysis of the temperature-dependent phase velocity variation is based on Figs 4 and 5. The waterfall diagram in Fig. 4 shows the changes of the antisymmetric wave mode at 40 kHz in response to temperature variations. This graph depicts the wave’s maximum where the delay in TOF changes linearly with temperature. The corresponding variations in TOF as a function of temperature are plotted in the left part of Fig. 5. In a next step, the changes of phase velocity have been computed based on the following relationship:

1 $${c}_{ph}(T)=\frac{\Delta X}{{t}_{0}+\frac{dt}{dT}\Delta T},$$

where Δ*X* is the distance between actuator *T*_4_ and receiver *T*_10_ (for this example Δ*X* = 0.44 m). The initial TOF *t*_0_ is computed by the cross-correlation of the actuation pulse and the received signal. Δ*T* is the temperature difference and $\frac{dt}{dT}$ the change of TOF with temperature. The latter has been extracted from the linear fit and is given by 0.2749 *μ* s/°C. The corresponding variations of the phase velocity with temperature is shown on the right side of Fig. 5 and ranges from 1166 m/s at 20 °C to 1133 m/s at 60 °C. This represents a phase velocity change with temperature of −0.825 m/s/°C which is in line with predictions from the literature^3^.

### Analysis of damage detection performance

#### Optimal Baseline Selection (OBS) and Baseline Signal Stretch (BSS)

**Fig. 6:**
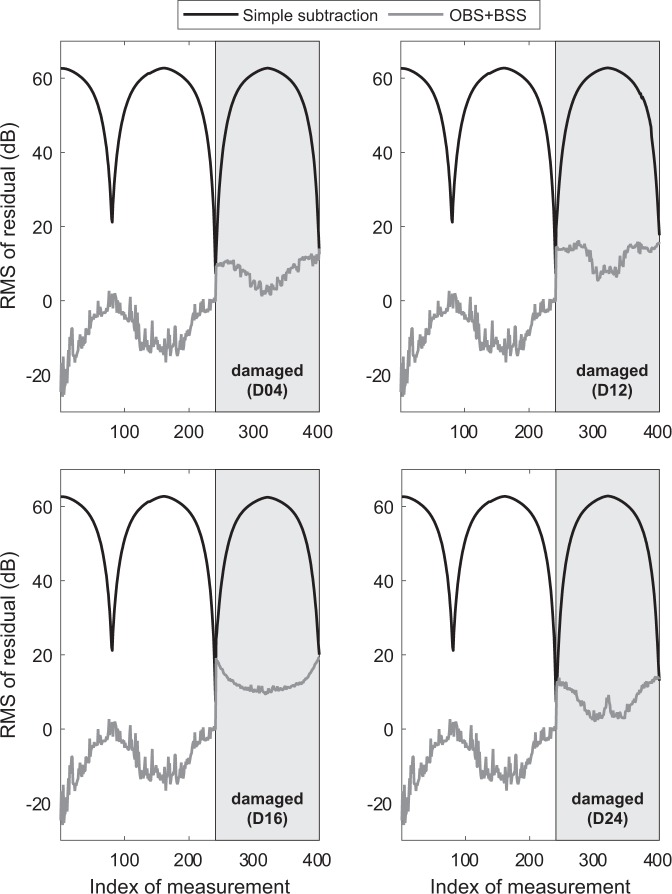
Damage detection result for the damage positions given in Table 2 using simple subtraction and temperature compensation (combined OBS + BSS strategy). Damage can only be detected in all four cases when the temperature influence was compensated.

Figure 6 shows the results for damage detection under variable temperature conditions where the simple subtraction approach is compared with the case in which the temperature effect was removed by the combined OBS + BSS strategy^5^ where OBS employs the aforementioned training set of baselines that are exposed to varying temperature. The simple subtraction method is not able to identify the surface mounted damage at a carrier frequency of 40 kHz. On the other hand, all four considered damage cases where successfully identified. It should be noted that the employed methodology has limitations for the detection of the surface mounted damage at higher ultrasound frequencies, because the out-of-plane component of the considered guided wave modes decreases towards higher frequencies.

#### Independent component analysis (ICA)

Given repeatedly over time acquired signals where OBS has been used for temperature-compensation, ICA seeks at finding a separation of the multivariate data *x* into maximally independent components *s*. Damage assessment can be performed through detecting changes in the strength *A* of the components over time. Formally, the relationships reads as *x* = *A* · *s*. However, commonly the data is mean-centered as well as whitened to have a covariance of unity *z* = *V* · *x*. The task is to find weights *w* such that *y* = *w*^*T*^ · *z* and its differential entropy *J*(*y*) becomes maximal. Practical implementations replace *J* by an appropriate nonlinear function *G*. Here, the FastICA algorithm (Version 2.5, http://research.ics.aalto.fi/ica/fastica/) is used^23^ and *G* is chosen such that its derivative reads $G^{\prime} =g\propto tanh\left(y\right)$.

In this work, the ICA-based approach proposed in^8^ is compared to an approach applying Principal Component Analysis (PCA) instead of ICA. PCA is a widely-used method for dimensionality reduction^24^. In addition to the comparison between the ICA-based versus PCA-based approach, a further comparison is introduced: (i) The relative strength of components is normalized and summed up. This yields curves that are similar in nature to the results obtained for OBS + BSS. Moreover, (ii) the individual relative strengths obtained through the multivariate decomposition are fed into a binary clustering procedure in order to automatically decide if a damage is present. PCA-based results are deterministic whereas ICA-based results are stochastic and are therefore re-run 20 times. Similarity matrices are constructed using the relative strength of a single component as one dimension and the point in time of the measurement as a second dimension. Here, time is scaled to lie in the interval (0,1). A so-called sanity check probes if the clustering delivered a continuous assignment of two clusters that are therefore connected in time within one cluster. Average-linkage clustering is used. ICA and PCA are set to find 3 components. This choice is motivated by the assumption that there is one steady noise-induced component, one component related to the varying temperature and one emerging component related to damage. Implementations of PCA as well as average-linkage clustering corresponds to the respective algorithms available in MATLAB R2017b.

**Fig. 7:**
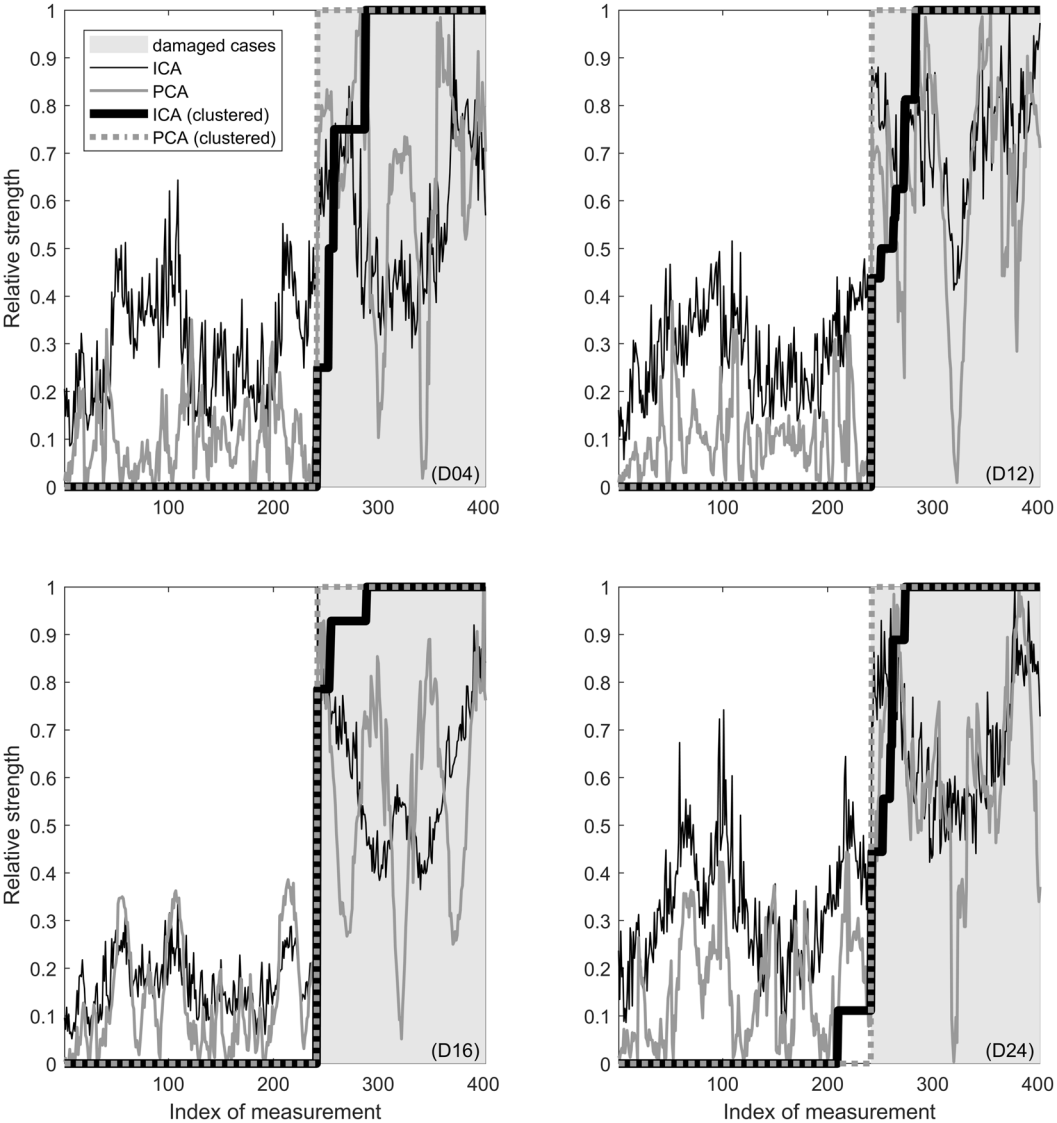
Damage detection results using ICA- and PCA-based methodology.

Figure 7 shows the results for damage detection where the aforementioned approaches are compared. The relative strengths for the intact cases are not always below the relative strengths for the damaged cases. However, the clustering approach is clearly able to discriminate between the pristine and defect plate. The PCA-based clustering approach even allows for error-free decision.

#### Singular value decomposition (SVD) and continuous baseline

As PCA can be done via an eigenvalue decomposition of the data covariance matrix, a singular value decomposition (SVD) can be used as well. In fact, the latter is numerically advantageous since formation of the covariance matrix can cause loss of precision. However, both are eigenvalue methods able to reduce high-dimensional datasets into less dimensions while retaining important information. Broadly speaking, PCA can be thought of as fitting the data by an *n*-dimensional ellipsoid, each axis representing a principal component. Similarly, the singular values of any (*m* × *n*)-data matrix *M* can be seen as the semi-axis of an *n*-dimensional ellipsoid in *m*-dimensional space. Therefore, $M=U\Sigma {V}^{* }$ for two square matrices *U*, *V* and a diagonal (*m* × *n*)-matrix Σ containing the singular values of *M*, which uniquely characterize its properties.

Performing SVD, e.g. using MATLABs built-in routine, for both baseline and the actual sensor signal, thereby reducing data dimensions tremendously, the discrepancy between the two corresponding singular values is calculated. Taking variations into account that do not correspond to a damaging effect, the baseline is continuously updated. This is based on the assumption that effects of damage occur suddenly (e.g. impacts), while environmental effects as well as aging act gradually, hence, resulting in a discrepancy that lies within a predefined tolerance. Whereas known techniques of continuous baseline growth^7^ extend a set of baselines in stock to improve OBS, here, the sole baseline is simply substituted, providing some sort of baseline-free acquisition. Thus, the algorithm makes the evaluation an evolutionary process, allowing for parallel acquisition and damage detection.

**Fig. 8:**
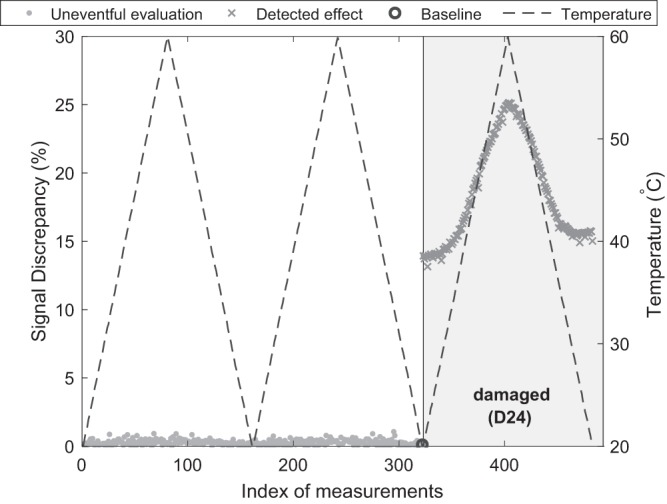
Damage detection result for path *T*_1_ to *T*_7_ at a carrier frequency of 40 kHz using evaluations based on SVD for updated baselines (here: exemplary for damage *D*_24_). Baseline continuously updates from first measurement until it detects the occurring damage. Then due to fixed baseline, evaluations vary with temperature.

Figure 8 depicts a result from such damage detection procedure. Therefore, recordings at sensor *T*_7_ of propagating waves excited from actuator *T*_1_ at a carrier frequency of 40 kHz were evaluated: at first two cycles of the undamaged structure with varying temperature, followed by one cycle with damage model *D*_24_. For the evaluating system the measurements appear to be acquired continually. The first baseline to begin with is the first measurement (assumed to represent an intact structure). As temperature changes gradually, the discrepancy of the next measurement is small within a predefined tolerance, such that the recording is categorized as ‘uneventful’ and becomes the actual baseline. Thus, gentle variance of the signal is compensated by an adaptive baseline. Only if the discrepancy is out of bounds, the baseline is not updated. Then, due to a baseline fixed at one temperature state, environmental changes affect the discrepancy as well.
